# Long noncoding RNA *MEG3* regulates LATS2 by promoting the ubiquitination of EZH2 and inhibits proliferation and invasion in gallbladder cancer

**DOI:** 10.1038/s41419-018-1064-1

**Published:** 2018-10-03

**Authors:** Longyang Jin, Qiang Cai, Shouhua Wang, Shuqing Wang, Tanmoy Mondal, Jiandong Wang, Zhiwei Quan

**Affiliations:** 1 Department of General Surgery, Xinhua Hospital, Shanghai Jiao Tong University School of Medicine, 200092 Shanghai, China; 2 Department of Medical Genetics, Institute of Biomedicine, The Sahlgrenska Academy, University of Gothenburg, SE-40530 Gothenburg, Sweden

## Abstract

Gallbladder cancer (GBC) is the most common type of biliary tract cancer worldwide. Long noncoding RNAs (lncRNAs) play essential roles in physiological and pathological development. LncRNA *MEG3*, a tumor suppressor, has been reported to play important roles in some cancers, but the role of MEG3 in GBC remains largely unknown. The purpose of the present study was to explore the role of MEG3 in proliferation and invasion and the potential molecular mechanism in GBC. We found that MEG3 was downregulated in GBC tissues and cells, and low expression of MEG3 was correlated with poor prognostic outcomes in patients. Overexpression of MEG3 inhibited GBC cell proliferation and invasion, induced cell apoptosis and decreased tumorigenicity in nude mice. Moreover, we found that MEG3 was associated with EZH2 and attenuated EZH2 by promoting its ubiquitination. Furthermore, MEG3 executed its functions via EZH2 to regulate the downstream target gene LATS2. Taken together, these findings suggest that MEG3 is an effective target for GBC therapy and may facilitate the development of lncRNA-directed diagnostics and therapeutics against GBC.

## Background

Gallbladder cancer (GBC) is the most common type of biliary tract cancer worldwide^1^. Despite the current advances in surgical therapy and chemotherapy for GBC, the overall 5-year survival rate for patients remains less than 5%^2^. Despite the potential for curative surgery, lymph node metastasis happens in 50% patients and less than 10% of tumors were resectable at the time of surgery^3^. Due to the rapid development of tumor biology and sequencing techniques, molecular pathogenesis has recently been recognized deeply for GBC^4^. Therefore, it is important to explore more molecular mechanisms for diagnosis and treatment in GBC.

It has been demonstrated that the majority of the human genome is transcribed into noncoding RNAs^5^. LncRNAs are a class of noncoding transcripts longer than 200 nucleotides that play essential roles in physiological and pathological development. LncRNAs execute their molecular functions as signals, decoys, guides, and scaffolds to realize biological outcomes^6^. Recently, more studies have found that lncRNAs are involved in modulation of many malignant cellular processes. For example, the lncRNA *HOTAIR* plays important roles in tamoxifen resistance in breast cancer^7^; the lncRNA *CAI2* contributes to the paradoxical overexpression of p16 and is associated with poor clinical outcomes in neuroblastoma^8^; and lncRNA *PCAT-1* decreases the tumor suppressor *BRCA2 in* prostate cancer^9^. Our previous study revealed that the lncRNA *UCA1* accelerated GBC progression by decreasing the transcription of the tumor suppressors p21 and E-cadherin^10^. Hence, it is essential to identify more cancer-associated lncRNAs and explore their biological functions and molecular mechanisms to provide new approaches for treatment of GBC.

In the present study, we demonstrated that another lncRNA, Maternally Expressed Gene 3 (MEG3), functioned as a tumor suppressor in GBC. MEG3 was downregulated in GBC tissues and cells, and its downregulation was related with poor prognosis in GBC patients. Furthermore, overexpression of *MEG3* inhibited GBC cell proliferation and invasion, induced cell apoptosis and decreased tumorigenicity in nude mice. We found that MEG3 was associated with EZH2 and degraded it through promoting its ubiquitination. Finally, MEG3 executed its function via EZH2 to regulate the downstream target gene Large Tumor Suppressor 2 (LATS2). In summary, our studies uncovered the MEG3-EZH2-LATS2 axis and may provide new strategies for diagnosis and treatment against GBC.

## Materials and methods

### Clinical data collection and GBC tissue specimens

Fifty paired GBC tissues and adjacent nontumor tissues were obtained from patients who underwent surgery at Xinhua Hospital (Shanghai Jiao Tong University School of Medicine, Shanghai, China) and Eastern Hepatobiliary Surgical Hospital and Institute (The Second Military Medical University, Shanghai, China) from 2009 to 2013. All tissues were stored in liquid nitrogen before RNA extraction. None of the patients received any local and systemic treatment before the surgery. All patients were staged according to the TNM staging system of the American Joint Committee on Cancer staging system. Complete clinicopathological data of every patient were collected. The present study was approved by the Human Ethics Committee of Xinhua Hospital, and informed consent was obtained from every patient.

### Cell lines and culture conditions

We used human GBC cell lines (NOZ, GBC-SD, SGC-996, EH-GB1, OCUG-1) and an immortalized human nontumorigenic biliary epithelial cell line (H69) in the present study. H69, GBC-SD, SGC-996, and OCUG-1 cells were purchased from the Cell Bank of the Chinese Academy of Science (Shanghai, China). NOZ was purchased from the Health Science Research Resources Bank (Osaka, Japan). EH-GB1 was received as a gift from Eastern Hepatobiliary Surgical Hospital and Institute. Five cell lines (H69, GBC-SD, SGC-996, EH-GB1, and OCUG-1) were cultured in DMEM high glucose medium (Gibco, USA), and NOZ was cultured in William’s Medium E (Genom, China) containing 10% fetal bovine serum (FBS, Gibco, USA) at 37 °C with 5% CO_2_.

### RNA extraction and qRT-PCR assays

Total RNA was extracted from tissue samples and cell lines with TRIzol reagent (Invitrogen, USA) according to the manufacturer’s protocol. Primer-Script One Step RT-PCR kit (TaKaRa, China) was used for reverse transcription. The SYBR Premix Dimmer Eraser kit (TaKaRa, China) was used for real-time RT-PCR. Primers were designed by Shanghai Sangon Biotech Co., Ltd., and are shown in Supplementary Table 1. β-actin expression was used for normalization. All the assays were performed in triplicate. The 2^–ΔΔCt^ method was used for calculation of the relative expression fold changes of RNAs.

### RNA interference

Small interfering RNAs and scrambled negative control (NC) siRNAs were used for transient transfection with Lipofectamine 2000 (Invitrogen), and the transfected cells were used after incubation for 48 h in assays. The siRNAs were synthesized by GenePharma (Shanghai, China). The siRNA sequences are displayed in Supplementary Table 1. Knockdown efficiencies were determined by qRT-PCR.

### Plasmid generation

The pcDNA-LATS2 vector was synthesized with the pcDNA3.1 vector and the LATS2 sequence for ectopic expression in cells. Negative control assays were performed with pcDNA3.1 vector. pCMV6-XL5-MEG3 was a generous gift from Tanmoy Mondal. Amplification efficiencies were determined by qRT-PCR.

### Cell counting kit-8 (CCK-8) assays

Cell proliferation was tested with a CCK-8 kit (Beyotime Institute of Biotechnology, China) according to the manufacturer’s instruction. Cells transfected with pCMV6-XL5-MEG3, pcDNA-LATS2 or NC vector and si-MEG3, si-LATS2 or si-NC were seeded into 96-well plates (1×10^3^ cells/well). We measured the absorbance at 450 nm every 24 h for 96 h. Each assay was performed in five replicate wells, and all the assays were conducted in triplicate.

### Flow cytometric analysis

After the transfection with the desired plasmid, siRNAs or si-NC and being incubated for 48 h, cells were harvested for analysis. The cells were fixed in prechilled 70% ethanol for 16 h at 4 °C and then stained with propidium iodide for cell circle analysis. For cell apoptosis analysis, the FITC-AnnexinV Apoptosis Detection Kit (BD Biosciences) was used according to the manufacturer’s protocol. For the mitochondrial membrane potential (mtΔΨ) analysis, the Mitochondrial membrane potential assay kit with JC-1 (Beyotime Institute of Biotechnology, China) was used according to the manufacturer’s protocol. In cells with high mtΔΨ, J-aggregates formed red fluorescence; while in cells with low mtΔΨ, the JC-1 monomer formed green fluorescence. The value of mtΔΨ was expressed as the ratio of red fluorescence intensity over green fluorescence intensity. All experiments were performed in triplicate.

### Colony formation assay

After being incubated for 48 h, the transfected cells were seeded into six-well plates (200 cells/well), and then the cells were incubated in incubator with 5% CO_2_ at 37 °C. The cells were fixed with 4% paraformaldehyde and then stained with 0.1% crystal violet after 2 week’s incubation. The numbers of the colonies were counted by visual inspection.

### Cell invasion assay

Twenty-four-well transwell invasion plates (Corning, USA) were used for cell invasion assays. The transwell plates were precoated with Matrigel (BD, USA). We seeded approximately 2×10^5^ transfected cells into the upper chamber with serum-free medium. The lower chamber had 500 μL medium with 10% FBS to stimulate invasion. After incubation for 24 h and removing the cells above the Matrigel, the cells that invaded the bottom chamber were fixed with 4% paraformaldehyde and stained with 0.1% crystal violet. Cells from five randomly chosen fields were counted.

### Western blotting

We first separated protein lysates with 15% SDS-PAGE and then transferred them to 0.22-mm NC membranes. After incubation with primary antibodies overnight at 4 °C, the membranes were incubated with horseradish peroxidase-labeled secondary antibody. All experiments were performed in triplicate.

### RNA immunoprecipitation (RIP)

The Magna RIP RNA-Binding Protein Immunoprecipitation Kit (Millipore, USA) was used for RIP experiments according to the manufacturer’s protocol. Anti-IgG and anti-SNRNP70 were used as the NC and positive control, respectively. The EZH2 antibody for RIP assays was purchased from Cell Signaling Technology.

### Chromatin immunoprecipitation (ChIP)

The EZ-CHIP KIT (Millipore, USA) was used for ChIP assays according to the manufacturer’s protocol. EZH2 antibody and H3 trimethyl Lys 27 antibody were purchased from Cell Signaling Technology and Millipore, respectively. Normal IgG antibody was used as the NC. Specific primers for LATS2 promoter are as follows: forward, 5′-ACCCCAAAGTTCGGACCTTAT-3′; reverse, 5′-CATTTGCCGGTTCACTTCTGC-3′. The results were calculated as a percentage relative to the input DNA.

### Ubiquitination assay

After centrifugation at 12,000 × *g* for 10 min at 4 °C, the supernatants of cell lysates from transfected cells were incubated with EZH2-specific antibody and protein A-Sepharose beads (Santa Cruz Biotechnology, USA) overnight at 4 °C. After being washed, precipitated proteins were boiled and then separated with SDS-PAGE. The monoclonal antibody against ubiquitin (Proteintech, USA) was used to determine the ubiquitination levels of EZH2.

### Tumor xenograft experiment

For stable overexpression of MEG3 in vivo, we first cloned the lncRNA-MEG3 gene into the lentiviral vector LV5-EF1aF-GFP/Puro (GenePharma, China), and LV-NC was transfected simultaneously as an NC. Four-week-old male athymic BALB/c nude mice were maintained under specific-pathogen-free conditions for tumor xenograft experiments. NOZ cells (100 μl, 1×10^6^) transfected with LV-MEG3 or LV-NC were injected subcutaneously in the left flank. Tumor size was monitored every week and calculated as 0.5 × length × width^2^. After 4 weeks, all mice were killed, and tumors were weighed and processed for immunohistochemical staining of Ki67 expression. The study was approved by the Animal Care and Use Committee of Xinhua Hospital.

### Immunohistochemistry

After all the mice were killed, the tumor tissues were fixed in 4% paraformaldehyde and embedded in paraffin. Then, 3-μm tumor sections were incubated with Ki67 antibody (CST, USA) at 4 °C overnight. The sections were then treated with secondary antibody for 30 min and stained with diaminobenzidine. All fields were assessed by two pathologists blindly under a light microscope.

### Statistical analysis

All statistical analyses were performed using SPSS 20.0. The associations between MEG3 expression and clinical features were analyzed by Pearson chi-square tests. We used paired samples *t* tests to analyze the expression differences of MEG3 between GBC tissues and the adjacent nontumor tissues. We also used independent samples *t* tests to analyze the expression differences between groups. Survival curves were calculated by the Kaplan–Meier method with the log-rank test. Univariate and multivariate Cox proportional hazards models were used to analyze the prognostic factors. Two-sided *p* values were calculated, and it was considered statistically significant when a *p* value was less than 0.05.

## Results

### MEG3 was downregulated in GBC tissues, and its low expression was associated with poor prognosis of GBC

**Fig. 1:**
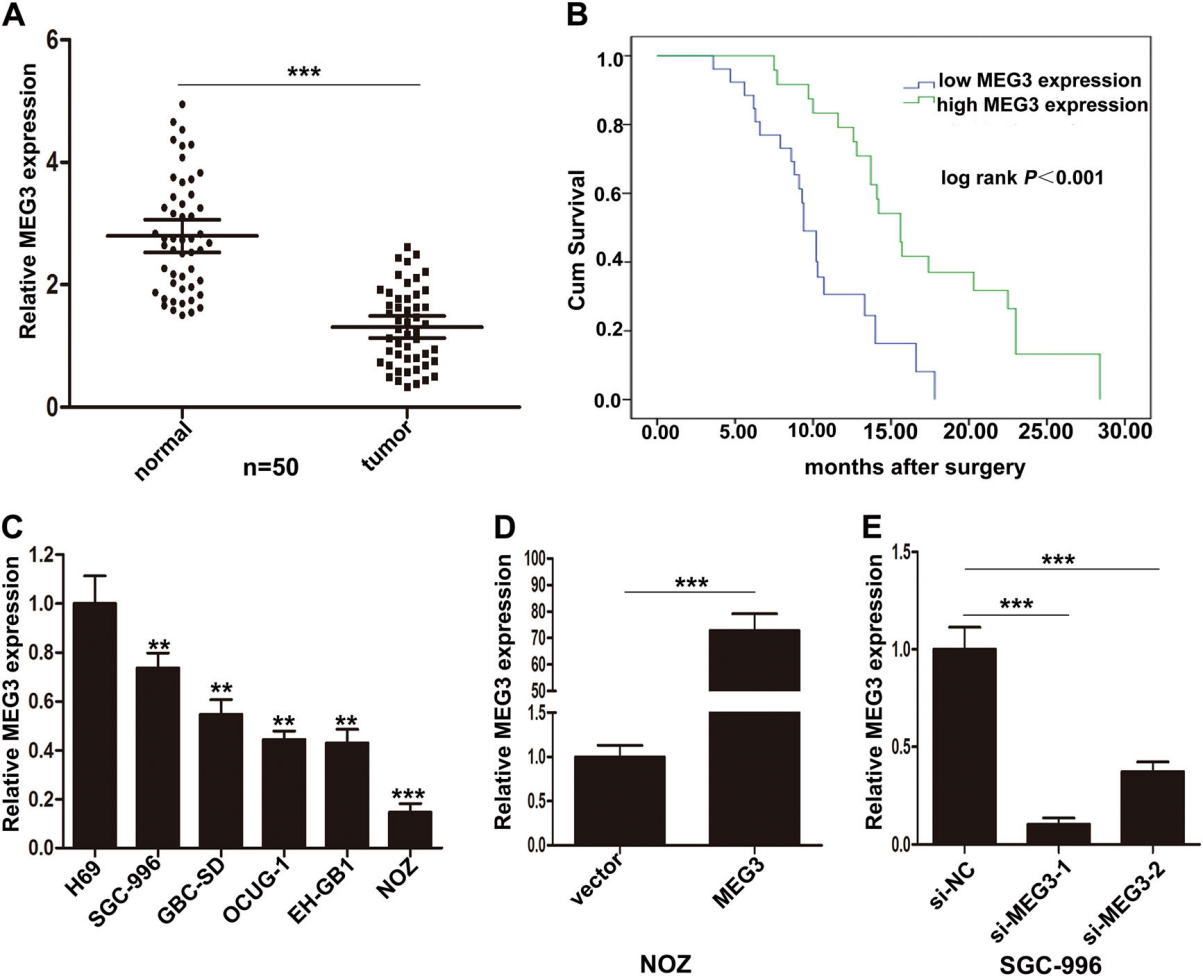
Relative expression of MEG3 in GBC tissues, cells and its clinical significance. **a** Relative expression of MEG3 in GBC tissues and paired neighboring nontumor tissues (*n* = 50). MEG3 expression was examined by qRT-PCR assays. **b** Kaplan–Meier analysis of overall survival according to MEG3 expression levels. **c** Relative expression of MEG3 in GBC cell lines and human biliary epithelium cell line H69 was detected by qRT-PCR. **d** Relative expression of MEG3 in NOZ cells transfected with MEG3 plasmid. **e** Relative expression of MEG3 in SGC-996 cells transfected with siRNAs. **p < 0.01, ***p < 0.001

To investigate the expression of MEG3 in GBC tissues and normal tissues, we assessed the MEG3 levels in 50 pairs of GBC tissues and adjacent nontumor tissues by qRT-PCR. The expression levels of MEG3 were significantly lower in GBC tissues than the adjacent nontumor tissues (Fig. 1a). Moreover, to analyze the relationship between MEG3 expression and clinicopathologic features, we divided 50 GBC patients into two groups according to the expression level of MEG3: the low MEG3 group (*n* = 26, fold change < mean ratio) and the high MEG3 group (*n* = 24, fold change ≥ mean ratio). It was found that low MEG3 expression was related with lymph node metastasis, histological grade, and TNM stage in GBC patients. However, MEG3 expression level was not associated with gender, age, and tumor size (Table 1).

**Table 1:** Correlation between MEG3 expression and clinicopathologic features of GBC patients

Characteristics	Case number	MG3 expression		*p* value
		High (*n* = 24)	Low (*n* = 26)	
Gender				0.76
Male	15	8	7	
Female	35	16	19	
Age				0.39
≤60	29	12	17	
>60	21	12	9	
Tumor size				0.159
≤5 cm	30	17	13	
>5 cm	20	7	13	
Lymph node metastasis				0.018*
Yes	32	11	21	
No	18	13	5	
Histological grade				0.046*
Well and moderately	23	15	8	
Poorly and others	27	9	18	
TNM stage				0.047*
I−II	12	9	3	
III–IV	38	15	23	

*p < 0.05

Furthermore, Kaplan–Meier survival analysis demonstrated that patients with low MEG3 levels had a shorter survival than those with high levels (Fig. 1b). Univariate survival analysis showed that tumor size, lymph node metastasis, low MEG3 expression, and TNM stage were prognostic factors. Moreover, multivariate Cox regression analysis showed that low MEG3 expression, along with TNM stage, was an independent prognostic factor for GBC patients (Supplementary Table 2).

### MEG3 was downregulated in GBC cell lines and affected cell proliferation and apoptosis in vitro

Furthermore, we evaluated the expression of MEG3 in five GBC cell lines (NOZ, GBC-SD, SGC-996, EH-GB1, OCUG-1) and an immortalized human nontumorigenic biliary epithelial cell line (H69) by qRT- PCR. The transcript levels of MEG3 were higher in H69 than GBC cells (Fig. 1c). For ectopic expression, we transfected pCMV6-XL5-MEG3 into the NOZ cell line, which had relatively low expression of MEG3, and we also designed two MEG3 siRNAs to transfect the SGC-996 cell line, which had relatively high expression of MEG3. QRT-PCR showed that MEG3 expression was significantly upregulated after transfection of the plasmid in NOZ, and si-MEG3-1 had the better interference efficiency in SGC-996 (Fig. 1d, e). Thus, we chose si-MEG3-1 for the following experiments.

**Fig. 2:**
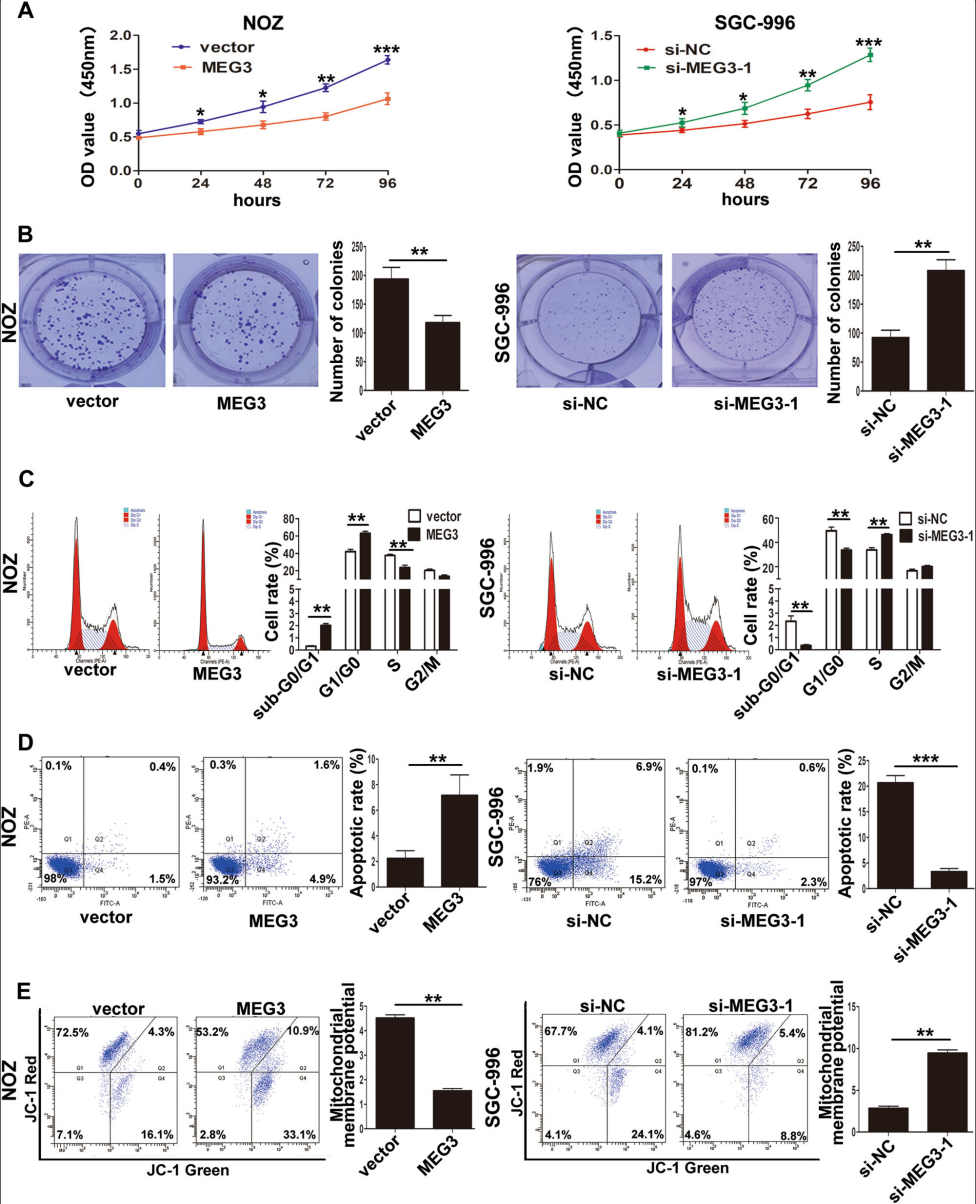
Effect of MEG3 on GBC cells growth in vitro. **a** The proliferation ability of NOZ cells transfected with MEG3 plasmid and SGC-996 cells transfected with si-MEG3-1 was determined by CCK8 assays. **b** The cloning ability of transfected NOZ and SGC-996 cells. **c** The cell cycle of transfected NOZ and SGC-996 cells. **d** The apoptosis of transfected NOZ and SGC-996 cells (Q1: AnnexinV-/PI+, Q2: AnnexinV+/PI+, Q3: AnnexinV−/PI−, Q4: AnnexinV+/PI−). **e** The mitochondrial membrane potential (mtΔΨ) analysis of transfected NOZ and SGC-996 cells. *p < 0.05, **p < 0.01, ***p < 0.001

To detect the effect of MEG3 on GBC cell proliferation, we performed CCK-8 assays. As shown in Fig. 2a, MEG3 overexpression significantly attenuated NOZ cell proliferation, while MEG3 knockdown promoted SGC-996 cell proliferation. Similarly, colony formation assays demonstrated that the clonogenic ability was impaired following MEG3 overexpression in NOZ cells, whereas MEG3 downregulation increased the clone-formation ability in SGC-996 cells (Fig. 2b).

**Fig. 3:**
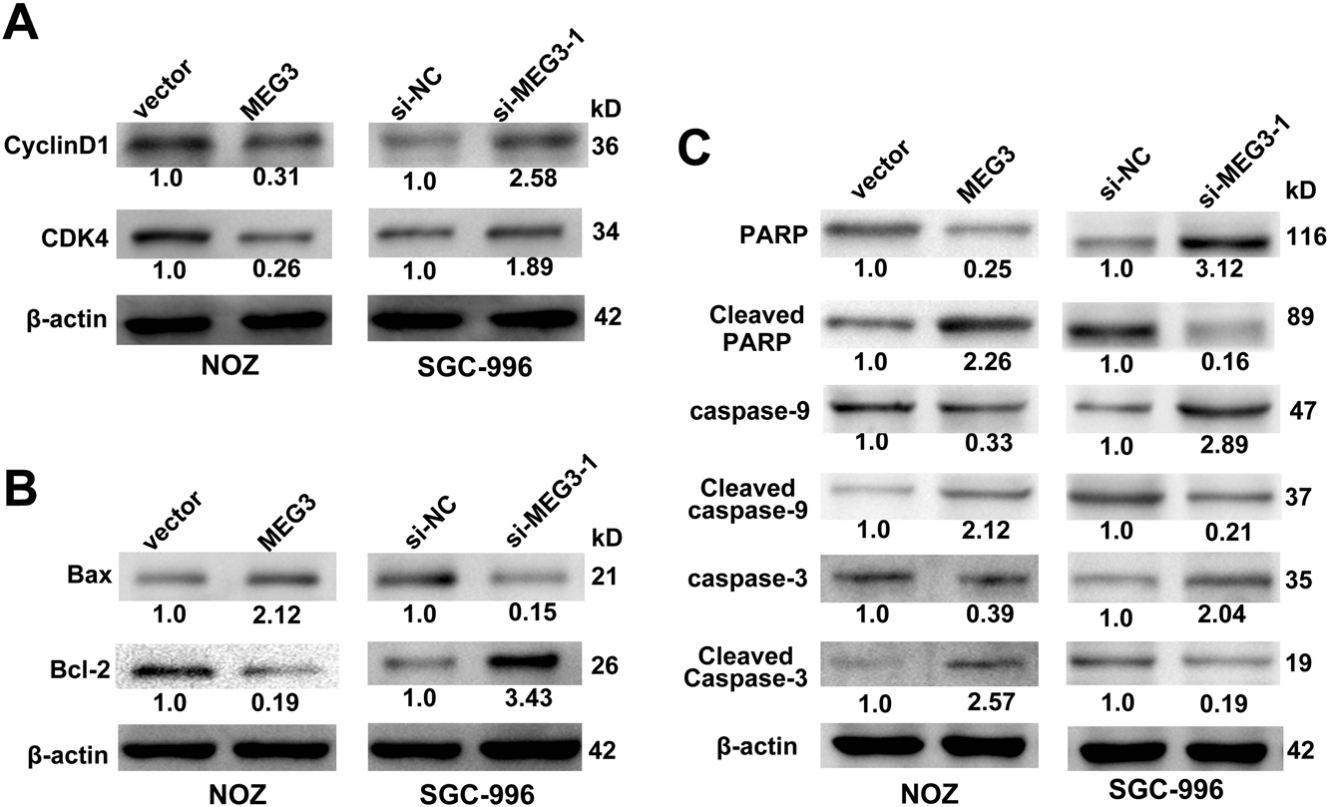
The proteins related with cell cycle and apoptosis induced by MEG3 were detected by western blotting assays. **a** The levels of CDK4, CyclinD1 protein in NOZ cells transfected with MEG3 plasmid and SGC-996 cells transfected with si-MEG3-1. **b**, **c** The expression levels of Bax, Bcl-2, Cleaved caspase-3, Cleaved caspase-9, and Cleaved PARP in transfected NOZ and SGC-996 cells

We then performed flow cytometry analysis to investigate the effect of MEG3 on the cell cycle and apoptosis. The results showed that MEG3 overexpression led to a significant G1/G0 phase arrest and induced the apoptosis of NOZ cells and vice versa in SGC-996 cells (Fig. 2c, d). To validate whether the mitochondrial pathway was involved in the apoptosis of GBC cells, the mitochondrial membrane potential (mtΔΨ) was measured and it showed that the mtΔΨ decreased in NOZ cells when MEG3 was overexpressed and vice versa in SGC-996 cells (Fig. 2e). Then, the expression levels of cell cycle- and apoptosis-related proteins were examined by western blotting. The results (Fig. 3a) showed that CDK4 and CyclinD1 were decreased in NOZ cells transfected with the MEG3 plasmid; increased expression of cleaved caspase-3, cleaved caspase-9, Bax, and cleaved PARP and decreased expression of Bcl-2 were also found (Fig. 3b, c) and vice versa in SGC-996 cells transfected with si-MEG3-1. These results suggested that MEG3 had tumor-suppressive effects that impeded proliferation and induced apoptosis of GBC cells.

### MEG3 inhibited GBC cell invasion and epithelial−mesenchymal transition (EMT) progression

**Fig. 4:**
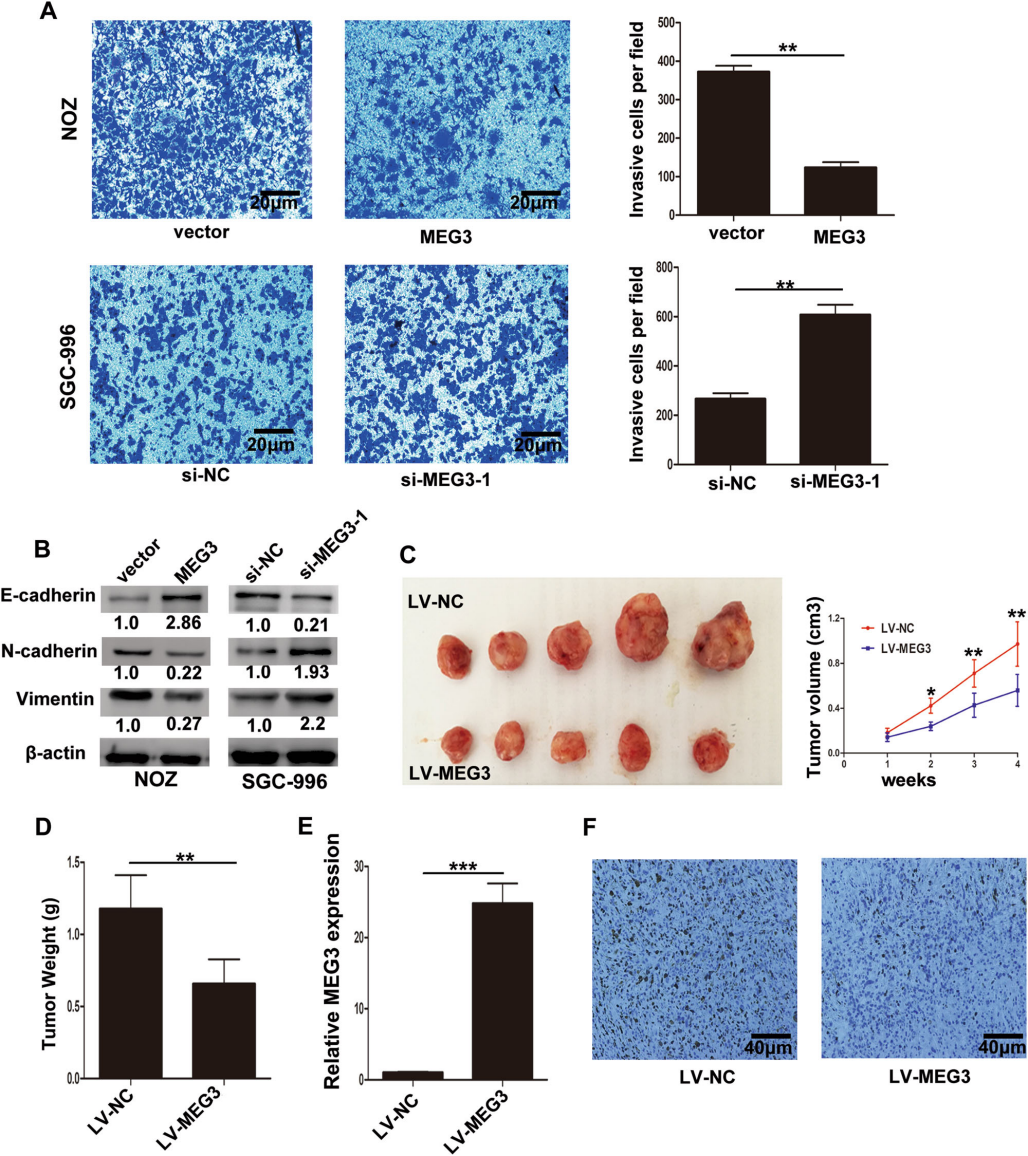
Effect of MEG3 on GBC cells invasion ability and tumor growth in vivo. **a** The invasion ability of transfected NOZ and SGC-996 cells was determined by transwell invasion assays. **b** The protein levels of E-cadherin, N-cadherin, and Vimentin in transfected NOZ and SGC-996 cells were determined. **c** The stable MEG3 overexpression NOZ cells were used for the in vivo study. The tumor volumes were measured per week until 4 weeks. **d** Tumor weight from LV-MEG3 and LV-NC groups was shown. **e** Relative expression of MEG3 in tumors from LV-MEG3 and LV-NC groups was detected by qRT-PCR. **f** The Ki67 expression of tumors from LV-MEG3 and LV-NC groups was determined by immunohistochemical staining. *p < 0.05, **p < 0.01, ***p < 0.001

Invasiveness is a significant feature of cancer cells, and to examine the effect of MEG3 on invasion of GBC cells, we conducted transwell invasion assays. MEG3 overexpression inhibited the invasion of NOZ cells compared with control cells, while knockdown of MEG3 increased the invasion of SGC-996 cells (Fig. 4a). EMT, a prominent feature of cancer, plays an important role in the progression of malignancy^11–13^. We proposed that MEG3 may also have effects on the EMT program based on the experimental results shown above. In addition, western blotting assays confirmed that NOZ cells transfected with the MEG3 plasmid exhibited a significant upregulation of E-cadherin, accompanied by downregulation of N-cadherin and Vimentin. Furthermore, the opposite results were detected after MEG3 knockdown in SGC-996 cells (Fig. 4b). These data indicated that MEG3 inhibited GBC cell invasion and EMT progression.

### MEG3 overexpression inhibited GBC cell tumorigenesis in vivo

To explore whether MEG3 could affect tumorigenesis in vivo, we transfected NOZ cells with LV-NC or LV-MEG3 and then injected them subcutaneously into nude mice. As shown in Fig. 4c, overexpression of MEG3 significantly inhibited tumor growth, and tumor weight was higher in the LV-NC group than the LV-MEG3 group (Fig. 4d). And qRT-PCR assays showed that the MEG3 levels in tumor tissues from the LV-MEG3 group were higher than those in tumors from the LV-NC group (Fig. 4e). Moreover, tumors from the LV-MEG3 group showed decreased Ki67 positivity compared with tumors from the LV-NC group (Fig. 4f). Taken together, these data indicated that upregulation of MEG3 inhibited tumor growth in vivo.

### MEG3 was associated with EZH2 and decreased its protein level by promoting ubiquitin–proteasomal degradation

**Fig. 5:**
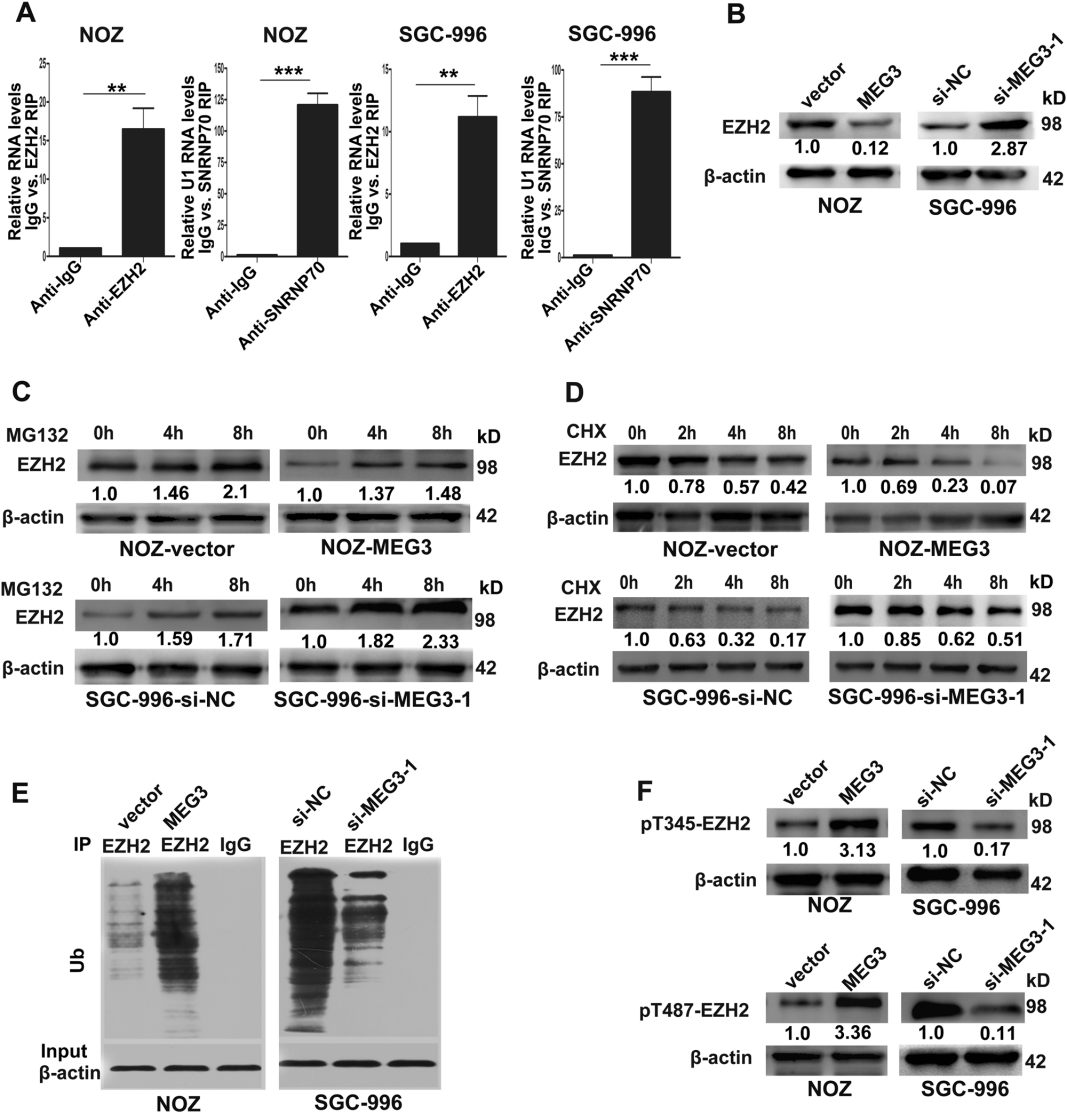
MEG3 was associated with EZH2 and promoted its ubiquitination and degradation by increasing EZH2 Thr-345 and Thr-487 phosphorylation. **a** Relative RIP assays detecting the binding of MEG3 with EZH2 in NOZ and SGC-996 cells by qRT-PCR. **b** The levels of EZH2 protein in NOZ cells transfected with MEG3 plasmid and SGC-996 cells transfected with si-MEG3-1. **c** The EZH2 protein expression was detected by western blotting in transfected NOZ and SGC-996 cells after treatment with MG132. **d** The EZH2 expression was detected in transfected NOZ and SGC-996 cells treated with CHX. **e** Western blotting of endogenous EZH2-associated ubiquitination in transfected NOZ and SGC-996 cells. **f** Western blotting of EZH2-Thr-345 and EZH2-Thr-487 phosphorylation in transfected NOZ and SGC-996 cells. **p < 0.01, ***p < 0.001

LncRNAs can compete with miRNAs^14,15^ or interact with RNA-binding proteins such as polycomb repressive complex 2 (PRC2)^16^ to regulate target genes. To explore the molecular mechanisms of MEG3 in GBC cells, we performed RIP to validate the association of MEG3 with EZH2 (the catalytic subunit of the PRC2) in NOZ and SGC-996 cells. The results showed that MEG3 could directly bind to EZH2 (Fig. 5a). Next, we conducted western blotting to characterize the effects of MEG3 on EZH2, and as shown in Fig. 5b, MEG3 overexpression significantly decreased EZH2 protein level in NOZ cells and vice versa in SGC-996 cells. Interestingly, we did not find any changes in EZH2 mRNA levels after the downregulation or upregulation of MEG3 (data were not shown). Therefore, we hypothesized that MEG3 could decrease EZH2 protein level after its transcription.

As many lncRNAs play roles in post-translational modifications of binding proteins^17,18^, a rationale for these results was that MEG3 may affect the stability of the EZH2 protein. To assess our hypothesis, we determined EZH2 expression in GBC cells incubated with the proteasome inhibitor MG132 and the protein synthesis inhibitor cycloheximide (CHX) after overexpression or knockdown of MEG3. As shown in Fig. 5c, EZH2 was modestly increased in GBC cells treated with MG132, indicating that MEG3 may degrade EZH2 protein by promoting the ubiquitin–proteasome pathway. Meanwhile, the CHX assay demonstrated that MEG3 could decrease the half-life of the EZH2 protein (Fig. 5d). We further performed ubiquitination assays to detect whether MEG3 could affect EZH2 protein stability and found that the EZH2 ubiquitination level was significantly increased in NOZ cells overexpressing MEG3, and the opposite phenomenon was found in SGC-996 cells after knockdown of MEG3 (Fig. 5e). These data indicated that MEG3 could attenuate EZH2 stability by promoting its ubiquitination.

Ubiquitination is a significant post-translational modification process in which the modified proteins are attached to the Ub, and their stability and functions in multiple cell processes and diseases, especially cancer development, are regulated^19,20^. Wu and Zhang^21^ found that the phosphorylation of EZH2 at the Thr-345 and Thr-487 sites promoted the ubiquitination of EZH2 and subsequent degradation by the ubiquitin–proteasome pathway. Therefore, in the present study, we conducted western blotting to detect the phosphorylation state of EZH2 at Thr-345 and Thr-487. As shown in Fig. 5f, MEG3 overexpression promoted the phosphorylation of EZH2 at Thr-345 and Thr-487 in NOZ cells, and the opposite result was observed when MEG3 was knocked down in SGC-996 cells. These data suggested that MEG3 promotes EZH2 ubiquitination by increasing phosphorylation of EZH2 at Thr-345 and Thr-487.

### MEG3 was required to promote EZH2 degradation to regulate LATS2 in GBC cells

**Fig. 6:**
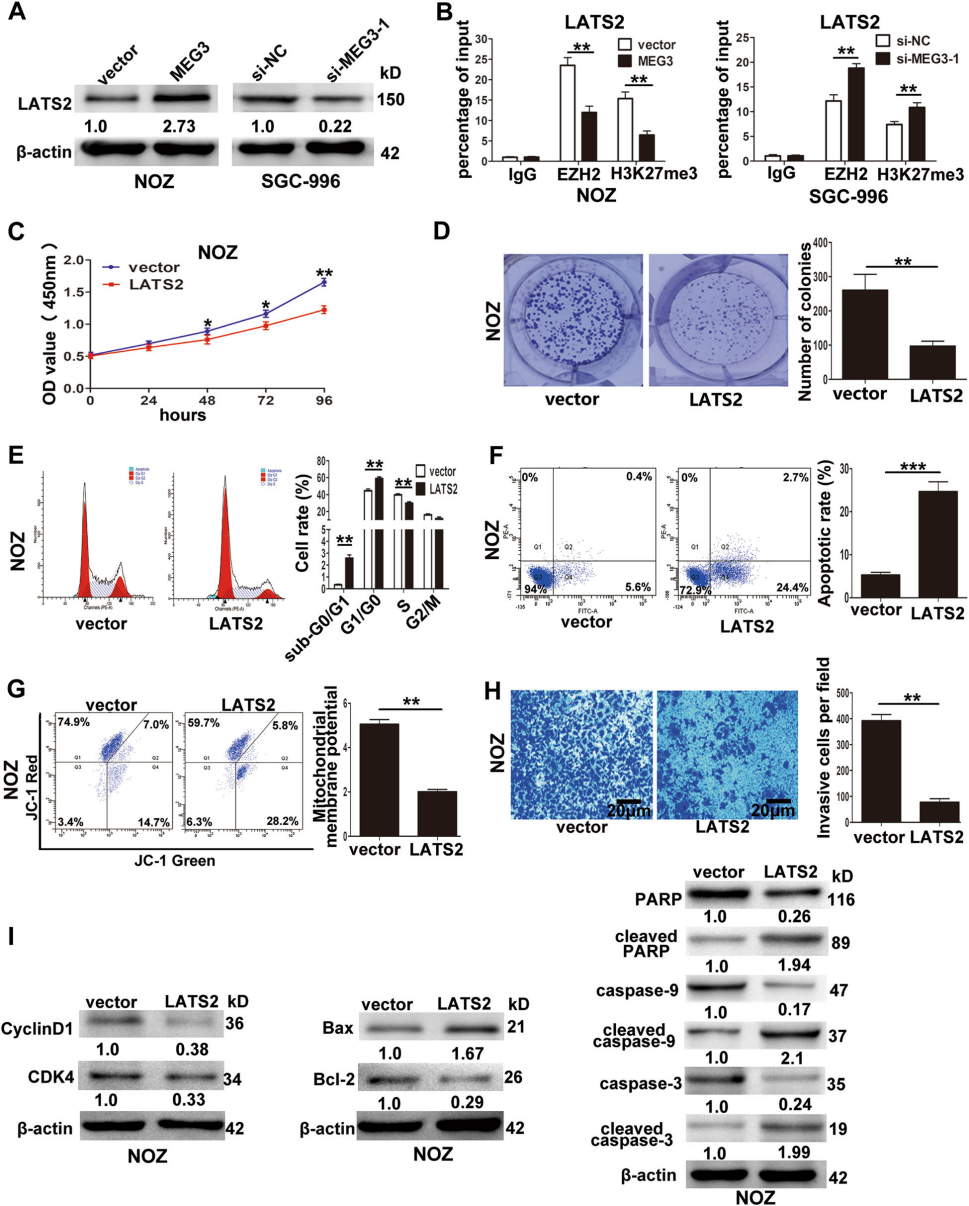
The association of LATS2 with MEG3, EZH2 and the effect of LATS2 on GBC cells growth in vitro. **a** The levels of LATS2 protein in NOZ cells transfected with MEG3 plasmid and SGC-996 cells transfected with si-MEG3-1. **b** ChIP-qRT-PCR analysis of EZH2 occupancy, H3K27me3 binding to the LATS2 promoter regions in NOZ and SGC-996 cells. **c** The proliferation ability of NOZ cells transfected with LATS2 plasmid determined by CCK8 assays. **d** The cloning ability of NOZ cells transfected with LATS2 plasmid. **e** The cell cycle of NOZ cells transfected with LATS2 plasmid. **f** The apoptosis of NOZ cells transfected with LATS2 plasmid. (Q1: AnnexinV−/PI+, Q2: AnnexinV+/PI+, Q3: AnnexinV−/PI−, Q4: AnnexinV+/PI−). **g** The mitochondrial membrane potential (mtΔΨ) analysis of transfected NOZ cells. **h** The invasion ability of transfected NOZ cells determined by transwell invasion assays. **i** The proteins related with cell cycle and apoptosis in NOZ cells transfected with LATS2 plasmid were detected by western blotting assays. *p < 0.05, **p < 0.01, ***p < 0.001

To explore the potential target genes of MEG3, we selected several EZH2 target genes with tumor suppressor functions that were identified previously (P15, P16, P21, P53, P57, E-cadherin, LATS2, PRSS8, KLF2, NKD2). QRT-PCR was performed to detect whether MEG3 overexpression had effects on these potential target genes, and we found that only the transcript levels of LATS2 were increased in NOZ cells transfected with the MEG3 plasmid (data was not shown). To further confirm that LATS2 was the downstream target of MEG3 in GBC cells, we performed western blotting. As shown in Fig. 6a, LATS2 protein level was increased in NOZ cells transfected with the MEG3 plasmid and decreased in SGC-996 cells transfected with si-MEG3-1. Next, we performed ChIP assays to confirm whether MEG3 regulated LATS2 via EZH2, and the results showed that EZH2 could directly bind to the promoter of LATS2 and induce histone H3 lysine 27 trimethylation (H3K27me3) in NOZ and SGC-996 cells (Fig. 6b). To further confirm that LATS2 was the downstream target of EZH2, we designed two siRNAs targeting EZH2, and qRT-PCR showed that si-EZH2-1 had higher knockdown efficiency. Western blotting showed that LATS2 protein levels were increased following EZH2 knockdown in NOZ and SGC-996 cells (Supplementary Figure 1A, B).

To detect the effect of LATS2 on GBC cells, we established pcDNA-LATS2 for ectopic expression in NOZ cells, and as shown in Supplementary Figure 1C, the mRNA level of LATS2 was significantly increased in NOZ cells after transfection of the plasmid. QRT-PCR showed that LATS2 overexpression had no influence on EZH2 mRNA and MEG3 (data were not shown). CCK-8 and colony formation assays demonstrated that LATS2 overexpression inhibited NOZ cell proliferation (Fig. 6c, d). Flow cytometry analyses showed that upregulation of LATS2 significantly increased G1/G0 phase arrest and promoted the apoptosis of NOZ cells (Fig. 6e, f). The mtΔΨ of NOZ cells also decreased when LATS2 was overexpressed (Fig. 6g). Transwell invasion assays demonstrated that LATS2 overexpression attenuated the invasion of NOZ cells (Fig. 6h). Furthermore, western blotting assays (Fig. 6i) showed that the cell cycle-related proteins CDK4 and CyclinD1 were downregulated, and the apoptosis-related proteins cleaved caspase-3, cleaved caspase-9, Bax, and cleaved PARP were upregulated, along with downregulation of Bcl-2, when LATS2 was overexpressed.

**Fig. 7:**
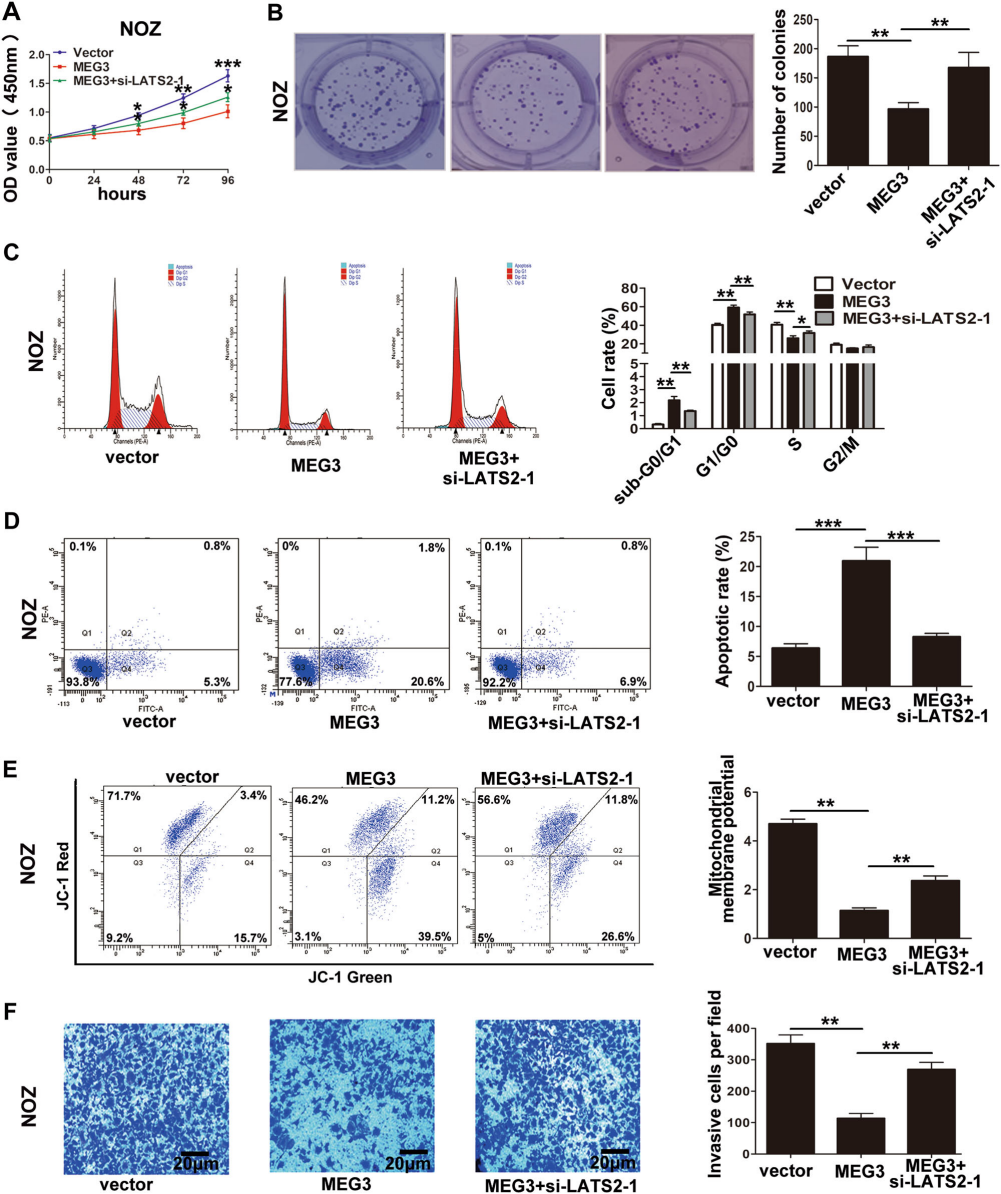
The proliferation and invasion results of NOZ cells cotransfected with MEG3 plasmid and si-LATS2. **a** The proliferation ability of NOZ cells cotransfected with MEG3 and si-LATS2 determined by CCK8 assays. **b** The cloning ability of NOZ cells cotransfected with MEG3 and si-LATS2. **c** The cell cycle of cotransfected NOZ cells. **d** The apoptosis of cotransfected NOZ cells (Q1: AnnexinV−/PI+, Q2: AnnexinV+/PI+, Q3: AnnexinV−/PI−, Q4: AnnexinV+/PI−). **e** The mitochondrial membrane potential (mtΔΨ) analysis of cotransfected NOZ cells. **f** The invasion ability of cotransfected NOZ cells was determined by transwell invasion assays. *p < 0.05, **p < 0.01, ***p < 0.001

Furthermore, to determine whether MEG3 regulated GBC cell proliferation and invasion via LATS2, we performed rescue assays. First, we designed two LATS2 siRNAs, and si-LATS2-1 had a higher efficiency as shown in Supplementary Figure 1D. Thus, we selected si-LATS2-1 for the following experiments. NOZ cells were cotransfected with the MEG3 plasmid and si-LATS2-1. CCK-8, colony formation, and flow cytometry analyses showed that si-LATS2-1 partially rescued MEG3-inhibited cell proliferation and reversed MEG3-induced cell apoptosis (Fig. 7a−e). Transwell invasion assays showed that si-LATS2-1 partially rescued MEG3-inhibited cell invasion (Fig. 7f). These data suggested that MEG3 played a role in GBC cells by promoting EZH2 degradation and then regulating LATS2.

## Discussion

In recent years, thousands of lncRNAs have been discovered by RNA sequencing and shown to play important roles in the development of disease, especially malignant progression^7–9^. Although alterations of many lncRNAs in GBC tumorigenesis have been studied^14,15,22^, the functional role and molecular mechanism of many GBC-associated lncRNAs remain unclear^23^.

*MEG3* is located at chromosome 14q32.3 in humans and functions as a novel lncRNA tumor suppressor^24^. MEG3 downregulation was shown to play important roles in promoting malignant transformation, accelerating cancer progression, and reducing chemosensitivity^25–27^. In the present study, we investigated MEG3 downregulation in GBC tissues and cell lines. Statistical analysis demonstrated that downregulation of MEG3 was correlated with lymph node metastasis, histological grade, and TNM stage and associated with poor prognosis in GBC patients. Moreover, MEG3 overexpression in NOZ cells inhibited cell proliferation and invasion and induced apoptosis. Furthermore, tumor xenograft assays confirmed that MEG3 amplification could decrease tumor growth in vivo.

EZH2, a subunit of PRC2, is a highly conserved histone methyltransferase and functions as a regulator to trigger H3K27me3 trimethylation and then represses the translation of target genes participating in numerous biological processes, including cell cycle regulation, senescence, cell proliferation, differentiation, apoptosis and tumorigenesis^28,29^. Many lncRNAs bind with EZH2 and then repress downstream genes^6,30^, and some lncRNAs can regulate target genes by influencing the stability of EZH2^31^. In the present study, we demonstrated that MEG3 could directly bind to EZH2 and regulate it at the post-translational level by promoting phosphorylation at Thr-345 and Thr-487 of EZH2, resulting in its ubiquitination and subsequent degradation.

LATS2, a member of the large tumor suppressor family^32^, has been confirmed to be a tumor suppressor through different signaling pathway in many cancers^33–35^. In our study, ChIP assays showed that EZH2 could directly bind to the promoter of LATS2 and induce H3K27me3 trimethylation in GBC cells. Moreover, LATS2 overexpression inhibited NOZ cell proliferation and invasion and induced apoptosis. Furthermore, rescue assays demonstrated that the effects of MEG3 were mediated through LATS2.

## Conclusions

In conclusion, the present study reported for the first time that the lncRNA MEG3 was downregulated in GBC tissues and cells, and low expression of this lncRNA may be a negative prognostic factor for GBC patients. MEG3 inhibited GBC cell proliferation and invasion, induced cell apoptosis and decreased tumorigenicity in nude mice. Moreover, MEG3 functioned by regulating the stability of EZH2, which suppressed the downstream gene LATS2. Our findings suggested that MEG3 is an effective target for GBC therapy and may facilitate the development of lncRNA-directed diagnostics and therapeutics against GBC.

## Electronic supplementary material

Supplementary Figure 1

Supplementary Table 1

Supplementary Table 2

Supplementary figure legends

## Data Availability

The datasets used in the current study are available from the corresponding author on reasonable request.

## References

[1] Zhu AX, Hong TS, Hezel AF, Kooby DA (2010). Current management of gallbladder carcinoma. Oncologist.

[2] Duffy A (2008). Gallbladder cancer (GBC): 10-year experience at Memorial Sloan-Kettering Cancer Centre (MSKCC). J. Surg. Oncol..

[3] Sheth S, Bedford A, Chopra S (2000). Primary gallbladder cancer: recognition of risk factors and the role of prophylactic cholecystectomy. Am. J. Gastroenterol..

[4] Kanthan R, Senger JL, Ahmed S, Kanthan SC (2015). Gallbladder cancer in the 21st century. J. Oncol..

[5] Djebali S (2012). Landscape of transcription in human cells. Nature.

[6] Wang KC, Chang HY (2011). Molecular mechanisms of long noncoding RNAs. Mol. Cell.

[7] Xue X (2016). LncRNA HOTAIR enhances ER signaling and confers tamoxifen resistance in breast cancer. Oncogene.

[8] Barnhill LM (2014). High expression of CAI2, a 9p21-embedded long non-coding RNA, contributes to advanced stage neuroblastoma. Cancer Res..

[9] Prensner JR (2014). PCAT-1, a long noncoding RNA, regulates BRCA2 and controls homologous recombination in cancer. Cancer Res..

[10] Cai Q (2017). Long non-coding RNA UCA1 promotes gallbladder cancer progression by epigenetically repressing p21 and E-cadherin expression. Oncotarget.

[11] Davis FM (2014). Induction of epithelial–mesenchymal transition (EMT) in breast cancer cells is calcium signal dependent. Oncogene.

[12] Chandrakesan P (2014). Utility of a bacterial infection model to study epithelial–mesenchymal transition, mesenchymal–epithelial transition or tumorigenesis. Oncogene.

[13] Tsuji T, Ibaragi S, Hu G (2009). Epithelial−mesenchymal transition and cell cooperativity in metastasis. Cancer Res..

[14] Ma MZ (2015). Long non-coding RNA CCAT1 promotes gallbladder cancer development via negative modulation of miRNA-218-5p. Cell Death Dis..

[15] Ma MZ (2016). Long noncoding RNA GCASPC, a target of miR-17-3p, negatively regulates Pyruvate Carboxylase-dependent cell proliferation in gallbladder cancer. Cancer Res..

[16] Li W (2016). Upregulated long non-coding RNA AGAP2-AS1 represses LATS2 and KLF2 expression through interacting with EZH2 and LSD1 in non-small-cell lung cancer cells. Cell Death Dis..

[17] Yang F (2013). Repression of the long noncoding RNA-LET by histone deacetylase 3 contributes to hypoxia-mediated metastasis. Mol. Cell.

[18] Yang F, Zhang H, Mei Y, Wu M (2014). Reciprocal regulation of HIF-1alpha and lincRNA-p21 modulates the Warburg effect. Mol. Cell.

[19] Voutsadakis IA (2013). Ubiquitin- and ubiquitin-like proteins-conjugating enzymes (E2s) in breast cancer. Mol. Biol. Rep..

[20] Zhou MJ, Chen FZ, Chen HC (2014). Ubiquitination involved enzymes and cancer. Med. Oncol..

[21] Wu SC, Zhang Y (2011). Cyclin-dependent Kinase 1 (CDK1)-mediated Phosphorylation of enhancer of Zeste 2 (Ezh2) regulates its stability*. J. Biol. Chem..

[22] Wang SH (2016). Long non-coding RNA MINCR promotes gallbladder cancer progression through stimulating EZH2 expression. Cancer Lett..

[23] Khandelwal A, Malhotra A, Jain M, Vasquez KM, Jain A (2017). The emerging role of long non-coding RNA in gallbladder cancer pathogenesis. Biochimie.

[24] Zhou Y, Zhang X, Klibanski A (2012). MEG3 noncoding RNA: a tumor suppressor. J. Mol. Endocrinol..

[25] Zhou C (2017). LncRNA MEG3 downregulation mediated by DNMT3b contributes to nickel malignant transformation of human bronchial epithelial cells via modulating PHLPP1 transcription and HIF-1α translation. Oncogene.

[26] Li L (2017). MEG3 is a prognostic factor for CRC and promotes chemosensitivity by enhancing oxaliplatin-induced cell apoptosis. Oncol. Rep..

[27] Zhang J, Lin Z, Gao Y, Yao T (2017). Downregulation of long noncoding RNA MEG3 is associated with poor prognosis and promoter hypermethylation in cervical cancer. J. Exp. Clin. Cancer Res..

[28] Sauvageau M, Sauvageau G (2010). Polycomb group proteins: multi-faceted regulators of somatic stem cells and cancer. Cell Stem Cell.

[29] Joshi P (2008). Dominant alleles identify SET domain residues required for histone methyltransferase of Polycomb repressive complex 2. J. Biol. Chem..

[30] Chen Q (2017). Long non-coding RNA NEAT1, regulated by the EGFR pathway, contributes to glioblastoma progression through the WNT/beta-Catenin pathway by scaffolding EZH2. Clin. Cancer Res..

[31] Li Z (2017). The degradation of EZH2 mediated by lncRNA ANCR attenuated the invasion and metastasis of breast cancer. Cell Death Differ..

[32] Visser S, Yang X (2010). LATS tumor suppressor: a new governor of cellular homeostasis. Cell Cycle.

[33] Gao Y (2017). Downregulation of MiR-31 stimulates expression of LATS2 via the hippo pathway and promotes epithelial-mesenchymal transition in esophageal squamous cell carcinoma. J. Exp. Clin. Cancer Res..

[34] Guo Y (2017). miR-302/367/LATS2/YAP pathway is essential for prostate tumor-propagating cells and promotes the development of castration resistance. Oncogene.

[35] Hoa L (2016). The characterisation of LATS2 kinase regulation in Hippo-YAP signalling. Cell. Signal..

