# Supplementary figures and images for: Long noncoding RNA MEG3 regulates LATS2 by promoting the ubiquitination of EZH2 and inhibits proliferation and invasion in gallbladder cancer
Source: Cell Death Dis. 2018 Oct 3;9(10):1017. doi: 10.1038/s41419-018-1064-1 (PMC6170488; doi:10.1038/s41419-018-1064-1)

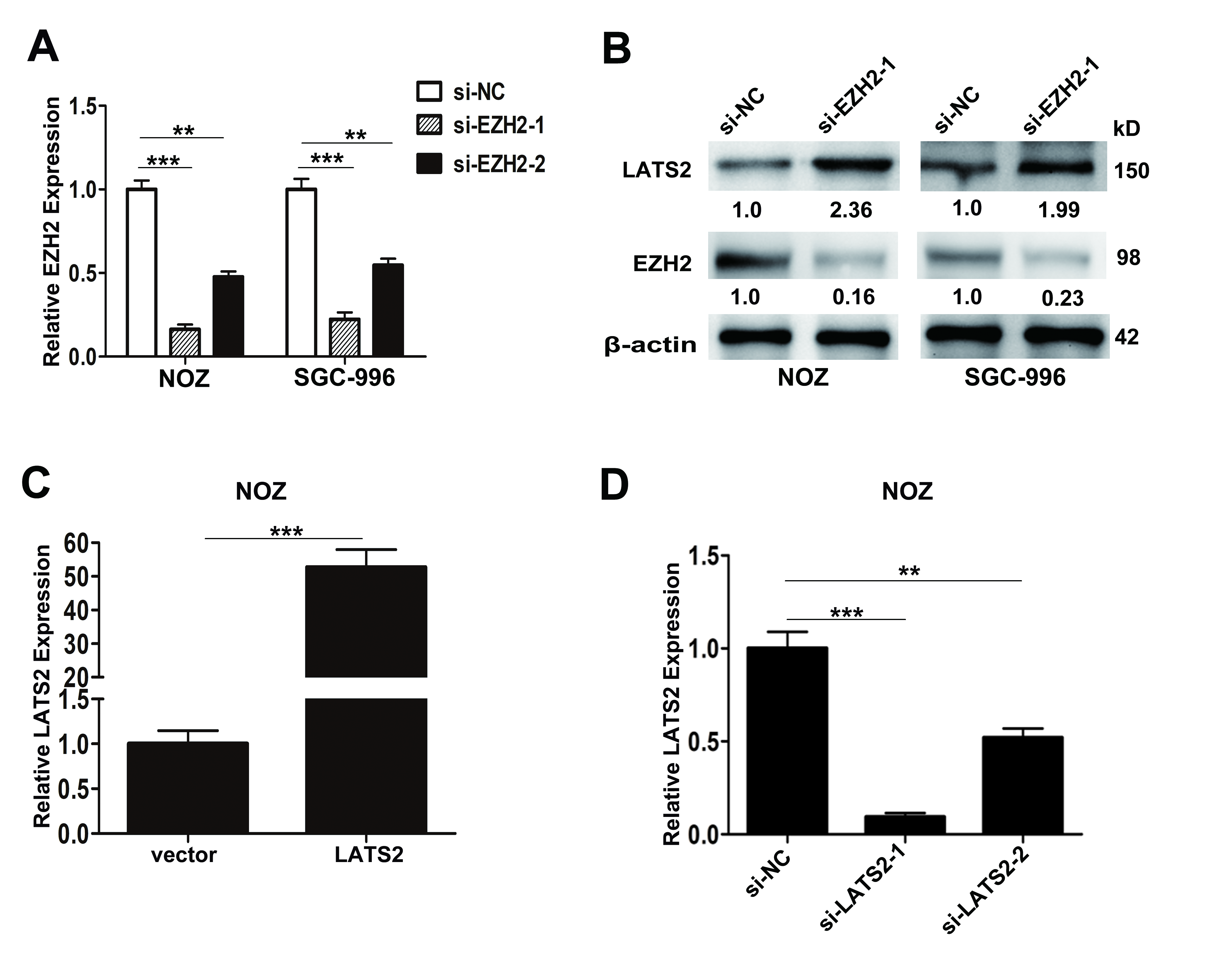

Supplement: Supplementary file 1 — Supplementary Figure 1 [file 41419_2018_1064_MOESM1_ESM.tif]
