# Supplementary material for: Long noncoding RNA MEG3 regulates LATS2 by promoting the ubiquitination of EZH2 and inhibits proliferation and invasion in gallbladder cancer
Source: Cell Death Dis. 2018 Oct 3;9(10):1017. doi: 10.1038/s41419-018-1064-1 (PMC6170488; doi:10.1038/s41419-018-1064-1)
Supplement: Supplementary file 3 — Supplementary Table 2 [file 41419_2018_1064_MOESM3_ESM.docx]

**Supplementary Table 2.** **Univariate and multivariate analysis of prognostic factors for overall survival in GBC patients.**

| Characteristics | **Univariate analysis** | |  | **multivariate analysis** | |
| --- | --- | --- | --- | --- | --- |
|  | **HR** | *P*-**value** |  | **HR(95%CI)** | *P*-**value** |
| **Gender** | 0.415 | 0.520 |  |  |  |
| **Age** | 1.235 | 0.266 |  |  |  |
| **Tumor size** | 4.060 | 0.044^*^ |  | 0.755(0.372-1.530) | 0.435 |
| **Lymph node metastasis** | 7.317 | ＜0.001^***^ |  | 0.391(0.148-1.037) | 0.059 |
| **Histological grade** | 0.899 | 0.343 |  |  |  |
| **TNM stage** | 8.550 | ＜0.001^***^ |  | 1.184(0.480-2.358) | ＜0.001^***^ |
| **MEG3 expression** | 7.695 | ＜0.001^***^ |  | 0.208(0.088-0.488) | ＜0.001^***^ |

**p*＜0.05， *** *p*＜0.001
