# Supplementary material for: Long noncoding RNA MEG3 regulates LATS2 by promoting the ubiquitination of EZH2 and inhibits proliferation and invasion in gallbladder cancer
Source: Cell Death Dis. 2018 Oct 3;9(10):1017. doi: 10.1038/s41419-018-1064-1 (PMC6170488; doi:10.1038/s41419-018-1064-1)
Supplement: Supplementary file 4 — Supplementary figure legends [file 41419_2018_1064_MOESM4_ESM.docx]

**FIGURE LEGEND**

**Supplementary Figure 1.** The amplification efficiency of LATS2 plasmid, the knockdown efficiencies of EZH2, LATS2 siRNAs and the effect of EZH2 on LATS2 protein. **A**, The knockdown efficiency of EZH2 si-RNAs in NOZ and SGC-996 cells. **B**, The expression levels of EZH2 and LATS2 protein after the knockdown of EZH2 in NOZ and SGC-996 cells. **C**, Relative expression of LATS2 in NOZ cells transfected with LATS2 plasmid. **D**, Relative expression of LATS2 in NOZ cells transfected with si-RNAs.
